# Multifunctional Smart Ball Sensor for Wireless Structural Health Monitoring in a Fire Situation

**DOI:** 10.3390/s20154328

**Published:** 2020-08-03

**Authors:** Minsu Kim, Insol Hwang, Minho Seong, Jaemook Choi, Myunggun Kim, Hee-Du Lee, Kyung-Jae Shin, Hungsun Son, Hoon Sohn, Junho Choi, Hoon Eui Jeong, Moon Kyu Kwak

**Affiliations:** 1 Department of Mechanical Engineering, Kyungpook National University, Daegu 41566, Korea; kms1685@knu.ac.kr (M.K.); mkkwak@knu.ac.kr (M.K.K.); 2 Department of Mechanical Engineering, Ulsan National Institute of Science and Technology (UNIST), Ulsan 44919, Korea; ihwang@unist.ac.kr (I.H.); sung710uio@gmail.com (M.S.); mgkim3070@unist.ac.kr (M.K.); hson@unist.ac.kr (H.S.); 3 Department of Civil and Environmental Engineering, Korea Advanced Institute of Science and Technology (KAIST), Daejeon 34141, Korea; cjmook@kaist.ac.kr (J.C.); hoonsohn@kaist.ac.kr (H.S.); 4 Department of Architectural Engineering, Kyungpook National University, Daegu 41566, Korea; lhdza@knu.ac.kr (H.-D.L.); shin@knu.ac.kr (K.-J.S.); 5 Department of Fire Protection Engineering, Pukyong National University, Busan 48513, Korea

**Keywords:** fire-induced collapse, fire situation, pre-engineered building (PEB), smart ball sensor, structural health monitoring (SHM)

## Abstract

A variety of sensor systems have been developed to monitor the structural health status of buildings and infrastructures. However, most sensor systems for structural health monitoring (SHM) are difficult to use in extreme conditions, such as a fire situation, because of their vulnerability to high temperature and physical shocks, as well as time-consuming installation process. Here, we present a smart ball sensor (SBS) that can be immediately installed on surfaces of structures, stably measure vital SHM data in real time and wirelessly transmit the data in a high-temperature fire situation. The smart ball sensor mainly consists of sensor and data transmission module, heat insulator and adhesive module. With the integrated device configuration, the SBS can be strongly attached to the target surface with maximum adhesion force of 233.7-N and stably detect acceleration and temperature of the structure without damaging the key modules of the systems even at high temperatures of up to 500 °C while ensuring wireless transmission of the data. Field tests for a model pre-engineered building (PEB) structure demonstrate the validity of the smart ball sensor as an instantly deployable, high-temperature SHM system. This SBS can be used for SHM of a wider variety of structures and buildings beyond PEB structures.

## 1. Introduction

In recent years, an increasing number of PEBs are being built mostly for industrial purpose due to the benefits that can be obtained from the cost efficiency and structural simplicity during the construction [1,2]. However, collapse vulnerability in a fire situation that arises from the structural simplicity of these structures has been a life-threatening factor to firefighters in an extinguishing action. Fire-induced fatalities and property damages are especially significant and frequent in PEBs [3,4].

Diverse sensors have been developed as a key element of structural health monitoring (SHM) systems for various buildings and infra structures, which include optical fiber sensors, triboelectric textile sensors, fiber Bragg grating sensors and surface acoustic wave sensors, piezoresistive sensors and cement-based strain sensors [5,6,7,8,9,10,11,12]. The SHM systems are essential to evaluate the safety, durability and lifecycle performance of buildings and civil structures. Many of these systems were developed for the operations at general temperature without significant considerations on high temperature conditions, such as a fire situation. Once fire occurs, structures are exposed to high temperatures of 300–1500 °C [13,14], which can induce structural collapses. Such fire-induced structural collapse has caused significant casualties and property damages [15]. However, most sensors used for the existing structural health monitoring system are not guaranteed under high-temperature environments. Moreover, the installation of the sensors on the specific point of structures in the event of a fire may not be practically possible.

Real-time data, such as vibration, temperature and relative displacement from the structure, can be utilized as indicators to predict structural health status and give alerts to firefighters to prevent possible casualties from the structural collapse [16]. For this purpose, highly durable SHM systems capable of stable data measurement and wireless data transfer even under fire conditions are prerequisite. Since installing sensors for SHM in all pre-existing buildings should cost extensive money and labor, it would be economically and practically favorable if a SHM system can be instantly installed on a pre-existing building in an emergency, especially such as a fire situation. However, despite recent developments of diverse SHM technologies, SHM systems that can be immediately installed onto target locations of structures and stably measure and transmit critical data from the structure in a fire situation have been barely explored.

Here, we report a deployable sensor system that can stably monitor structural health status in a fire and predict fire-induced structural failure and collapse by integrating sensors and wireless communication module, heat insulator and adhesive module in a light–weight and compact ball-type device configuration. With the integrated device configuration, the SBS can be easily installed on a target surface of structures on fire with strong adhesion and also stably measure vital structural vibration and temperature data in real time. The measured data could be stably transmitted to a receiver computer by using a wireless communication protocol. The effectiveness of the SBS as an instantly installable, fireproof SHM system was further demonstrated using a model PEB testbed.

## 2. Overall, Design and Working Principle of the Smart Ball Sensor

**Figure 1:**
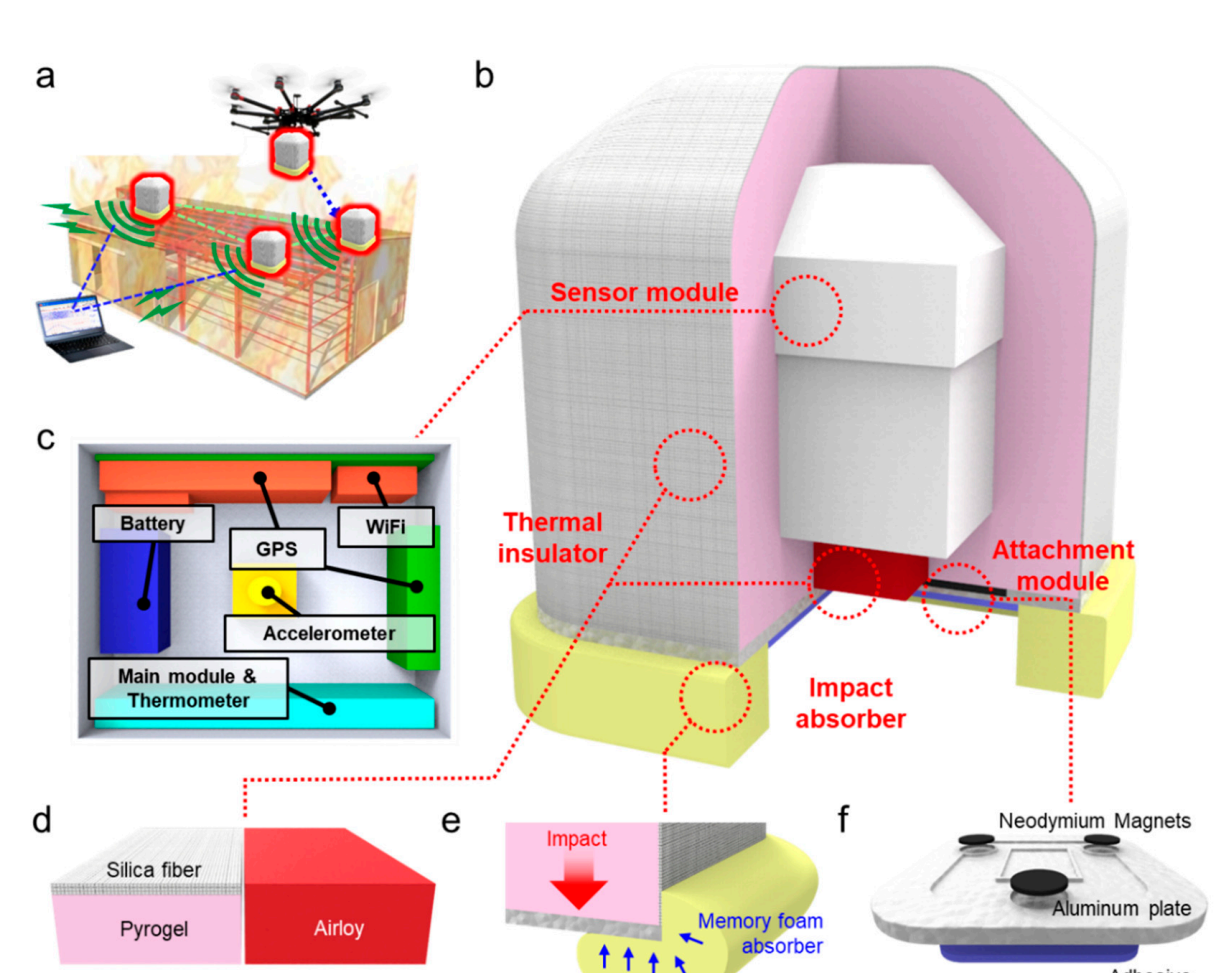
Conceptual drawing of remotely deployable smart sensor device for structural health monitoring system of pre-engineered building (PEB) structure in a fire situation. (**a**) Schematic of the smart ball sensor in a fire situation; (**b**) illustration of the smart ball sensor consisting of various module; (**c**) schematic of sensor module, (**d**) thermal insulator, (**e**) impact absorber and (**f**) attachment module.

According to case studies and our simulation results, when a fire occurred for PEB structures, cross beams supporting the roof first collapse [16]. Therefore, to predict structural deformation and collapse of PEBs, SHM systems need to be installed near the primary cross frames (roof beams) that support the PEB roof. Given that getting inside the PEB is difficult during a fire, the SBS needs to be installed above the roof beams. For this, we utilized a drone. Once per fire occurs, a drone carries SBSs and locates them on the designated locations of the PEB’s roof (Figure 1a). We designed the SBSs to have a compact size and light weight so that drones or firefighters can easily carry the SBSs and install them on the target locations of the PEB’s roof. The SBS has about 20 cm in diameter and 1.4 kg in weight. The SBS mainly consisted of sensor module, heat insulator, impact absorber and adhesive (Figure 1b). The sensor module had accelerometer, thermometer, Wi-Fi, battery and optional GPS (Figure 1c), which were fixed in a 3D printed polyamide (PA) case. To bear high temperature under a fire situation, the sensor module was passivated with heat insulator. The heat insulator was composed of Airloy, Pyrogel and silica fabric (Figure 1d). The shock absorber was installed on the surface of the bottom aluminum support of the SBS to protect the sensor module from physical impact (Figure 1e). The adhesive module consisted of high temperature inorganic adhesive and neodymium magnet (Figure 1f), which enables a firm attachment of the SBS on the PEB structure. Detailed structures and functions of each module are further described in the following sections.

## 3. Structure and Performance of the Sensor Module

**Figure 2:**
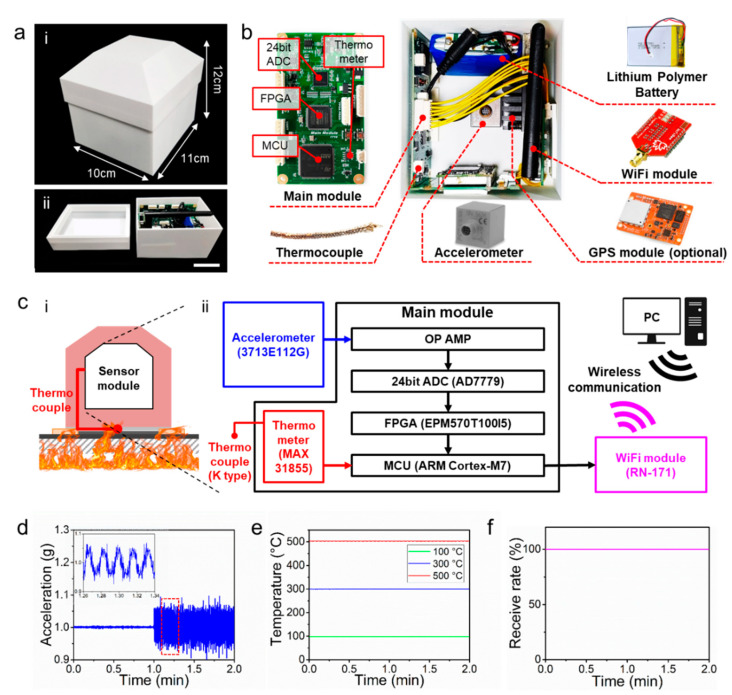
Configuration diagram of the sensor module and performance verification of each sensing device. (**a**) Photography of 3D printed sensor module case and its (i) dimensions and (ii) internal configuration (scale bar: 5 cm); (**b**) components of the sensor module; (**c**) conceptual drawing of (i) sensing in fire situation and (ii) schematic of sensor module and its wireless communication between the module and receiver; (**d**) performance evaluation of the accelerometer, (**e**) thermometer and (**f**) wireless transmission.

According to a previous report, a prediction of fire-induced structural failure and collapse based on visual imaging methods utilizing video camera and infrared camera have limited effectiveness in warning the onset of the collapse [17]. The time between the first appearance of structural deformation and the actual structural collapse is typically too short to warn the collapse. The presence of hot smoke plumes or water during the fire suppression hinder the accurate evaluation of the thermal signature of the structures. SHM methods based on the detection of structural vibrations could have the potential for more accurate diagnosis of structural status [17]. To this end, we integrated an accelerometer (3713E112G, PCB piezotronics) as a key element of the sensing module (Figure 2a,b). The detectable range of the accelerometer is ±2 g (0.1 mg rms) (Table 1). A thermocouple (Thermocouple K Type, Sparkfun) was extended through a hole of the sensing module’s case to the bottom side of the SBS so that it can measure the temperature of the contacting surface (Figure 2c(i)). The accelerometer and thermometer (MAX31855, Maxim integrated.) were connected to the mainboard, and the data were wirelessly transmitted to a receiver via a Wi-Fi module (RN-171, Roving networks) (Figure 2c(ii)). The two lithium polymer batteries (3.7 V, 900 mA, H503450, Z-SUN Technology Co.) were connected in series and used as a power source. A GPS can be installed into the sensing module as an optional element (Figure 2b).

**Figure 3:**
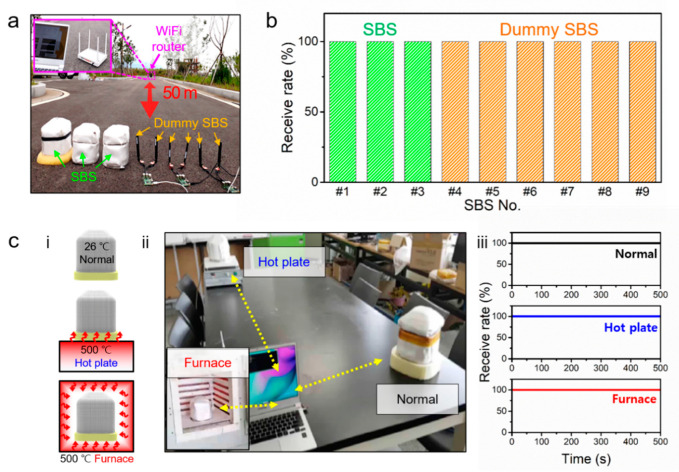
(**a**) Photograph of wireless communication test at a distance of 50 m; (**b**) data receive rate measured with three smart ball sensors (SBSs) and six dummy SBS sensors, showing that the thermal insulations of SBS do not hinder wireless communication and all SBS and dummy SBS shows 100% receive rate without each other’s interference; (**c**) schematic of (i) three different heating conditions and (ii) its photographic images of wireless communication test; (iii) Data receive rate shows normal wireless communication in heating conditions of normal (black), hot plate (blue) and furnace (red).

After preparing the integrated sensor module with the accelerometer, thermometer, Wi-Fi and battery, the performance of each element was evaluated. Figure 2d shows the acceleration data measured with the accelerometer after applying 1 Hz of periodic vibrations of 2 cm amplitude. The accelerometer could accurately detect the external vibrations with high accuracy. The acceleration value of 1 g before the application of periodic vibrations was caused by the gravity of Earth. The thermometer could perceive three different temperatures of 100 °C, 300 °C and 500 °C with high accuracy (error range: ±5 °C) (Figure 2e). The measured physical data were transmitted using a user datagram protocol(UDP)-wireless fidelity(Wi-Fi) protocol. Three SBS prototypes and six dummy SBSs were used to evaluate the wireless transmission ability of the sensing module. When the prototypes and dummies were 50 m away from the receiver, all of the nine SBS models exhibited an average data-receiving rate of 100% at the transmission speed of 100 data per seconds (Figure 2f, Figure 3a,b). The data transmission was successful even when the SBS prototypes were located in high temperature conditions due to the thermal insulating module of the SBS (Figure 3c).

## 4. Structure and Performance of the Thermal Insulator

**Figure 4:**
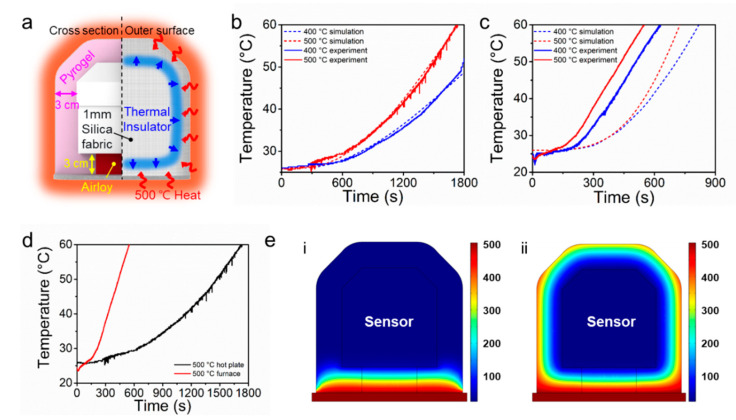
(**a**) Schematic of the insulating materials for thermal protection of SBS; (**b**) Experiment and simulation temperature of the sensor module when only the bottom of an SBS was heated on a 400–500 °C hot plate surface and (**c**) when the entire surfaces were heated by 400–500 °C air and bottom surface in furnace; (**d**) comparison of the experimental results between extreme and minimum conditions at 500 °C; (**e**) cross-section images acquired from the FEA result, showing the temperature distribution when the temperature of the sensor module reaches 60 °C, (i) on a 500 °C hot plate and (ii) in a 500 °C furnace.

Sensor packaging materials should have a low thermal conductivity to protect the sensor module from the intense heat of the surroundings. The densities of those materials should be low to reduce the total weight of the SBS. Moreover, the supporting material between the sensor module and target surface must be a hard, solid substance having a low damping coefficient to secure the maximum sensitivity of sensors, such as an accelerometer. Considering these requirements, we chose Pyrogel, Airloy and silica fabric as thermal insulating, packing materials to maximize the operating time of the sensor module at high temperatures (Figure 4a). Physical characteristics and thermal properties of the packaging materials are shown in Table 2. Pyrogel is a blanket type insulation material formed of silica aerogel and glass fiber. Silica aerogels have the lowest density (0.2 g cm^−3^) and thermal conductivity (0.025 W mK^−1^) among any known solids [18]. However, a practical application of silica aerogel is limited due to its high brittleness. With silica aerogels reinforced with nonwoven glass fiber batting, Pyrogel has great heat insulating performance in a flexible and easily applicable form [19]. The flexible characteristic of Pyrogel makes it easy to wrap the sensor module meticulously, whereas it is not preferred in regions between the module and target surface because any deformation of Pyrogel would directly result in degradation of the sensitivity of certain sensors, such as an accelerometer. Airloy is a mechanically robust polyimide aerogel that has a relatively high compressive strength (113 MPa). This material has a very low thermal conductivity (0.032 W mK^−1^); as such, the SBS can be protected from thermal damage without sacrificing sensor’s sensitivity by placing the Airloy tile between the sensor module and bottom aluminum support of the SBS.

The average thicknesses of the Pyrogel and Airloy used for the SBS were both 3 cm. The sensor module insulated with Pyrogel and Airloy was finally wrapped with the flexible silica fabric (thickness: 1 mm), which also has low thermal conductivity (0.033 W mK^−1^).

The fireproof performance of the SBS was evaluated by exposing the prepared SBS to elaborated temperatures. The temperature of the heating tests was set to 500 °C because the PEB structure collapses abruptly at that temperature [20,21]. By considering the maximum operating temperatures (guaranteed by the manufacturer) of each element of the sensor module (Table 1), we set the maximum allowable temperature inside the sensor module to 60 °C. The battery has the lowest operating temperature of 60 °C. As described above, the SBS would measure the physical quantities by being attached on the roof of the PEB. Accordingly, the test conditions were separated into extreme and minimum cases. The minimum condition assumed a fire situation in which only the bottom surface of the PEB roof is heated due to the fire inside the building. The extreme condition reflects a situation in which the sensor unit is surrounded by flames and the temperature rises along the entire surface of the SBS. For the minimum condition, the SBS was placed on a hot plate heated to 500 °C and the internal temperature was measured in real time by using a thermometer. According to the test results, the internal temperature reached 60 °C after 1724 s of heating (Figure 4b). This result indicates that the SBS can properly operate and detect the structural health status for 1724 s without system failure even under the fire situation. For the tests under the extreme condition, the SBS was placed in a heating furnace with heating temperature of 500 °C. The temperature data inside the sensor module was wirelessly collected. The internal temperature reached 60 °C after 548 s in the heating furnace (Figure 4c). Therefore, the SBS can maintain its monitoring function for 548 s minimum and 1724 s maximum in a 500 °C environment (Figure 4d). Based on discussion with firefighters, this time duration can be useful to alert structural collapse of the PEB to the firefighters. The time duration can be increased by using thicker thermal insulators upon requirements. Finite element analysis (FEA) of the internal temperature of the sensor module under the two different heating conditions were overall in good agreement with the experimental results (Figure 4b,c,e).

## 5. Structure and Performance of the Adhesive and Impact Absorber Modules

### 5.1. Attachment Module

**Figure 5:**
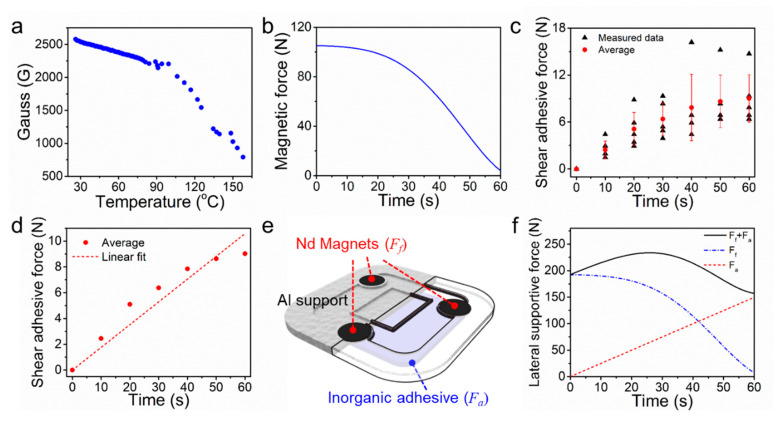
(**a**) Change in the magnetic flux density of Nd magnets according to the increasing temperature; (**b**) change in the magnetic force of an Nd magnet when it is heated on a 500-°C hot plate; (**c**) the maximum shear adhesive forces of five trials of the inorganic adhesive spread on an area of 7.07 cm^2^ (**d**) and its linear fitting; (**e**) schematic of the attachment module using three Nd magnets and the inorganic adhesive spread on an area of 100 cm^2^; (**f**) maximum static frictional force due to three Nd magnets (blue), the shear adhesive force of the inorganic adhesive spread on an area of 100 cm^2^ (red) and the total lateral supportive force (black).

The SBS is required to have a strong initial attractive force between the device and target surface while it retains a persistent force at a high temperature to ensure firm solid contact during the device’s operation to be able to deploy on a targeted surface of PEB from a distance and firmly fix on the surface. A neodymium (Nd) magnet can be an ideal candidate to give strong initial attractive force between the device and ferromagnetic PEB surface [22]. However, Nd magnets rapidly loose permanent magnetic properties when it is exposed to high temperatures due to its relatively low Curie temperature [23]. Indeed, the magnetic flux density of the Nd magnet (diameter: 50 mm, thickness: 5 mm) placed on a hot plate decreased with increasing heating temperature (Figure 5a). When a Nd magnet was placed on a hot plate with heating temperature of 500 °C, the magnetic force of a single Nd magnet (***F_m_***) against a steel plate was reduced from 105.2 N to 4 N (Figure 5b) after 60 s of heating. However, the magnet maintained fairly high magnetic forces during the initial 0–30 s. This result indicates that the Nd magnet can be utilized as an initial attachment mechanism of the SBS on the heated PEB surface. The lateral supportive force ***F_f_*** by magnets can be obtained by the equation ***F_f_ = n(μF_m_)***, in which ***n*** is the number of magnets used for the SBS and ***μ*** (aluminum–steel: 0.61) is the frictional coefficient.

In contrast to the magnet that loose adhesive force at high temperatures, an inorganic adhesive, which consists of silica and alumina, can exhibit a strong, prolonged adhesive force at high temperatures up to 1100 °C [24]. However, a certain time duration is required for the inorganic adhesive to be dried sufficiently to exhibit adhesion forces for the stable fixation of the SBS. Figure 5c shows the shear adhesive force of an inorganic adhesive as a function of heating time at a fixed heating temperature of 500 °C. For the adhesion tests, 0.30 g of the adhesive putty (CEMEDINE, Japan) was spread over the area of 3 cm diameter between two stainless steel pieces (spread area: 7.07 cm^2^). Considering the large deviation of the measured shear adhesive force of the inorganic adhesive, we used the average values among the measured values from five trials. As shown in Figure 5d, the shear adhesive force of the inorganic adhesive (***F_a_***) increased with heating time (1.52 N/cm^2^ after 60 s), which indicates that the inorganic adhesive can exert enough adhesion for the stable fixation of the SBS on the PEB once a certain time is allowed for its drying.

By considering the different temperature-dependent adhesion behavior of the magnet and the inorganic adhesive, stable fixation of the SBS on a high-temperature PEB surface could be achieved by integrating the strong initial adhesion force of the magnet and long-lasting adhesion force of the inorganic adhesive in a complementary way (Figure 5e). The total lateral supportive force (***F_f_*** + ***F_a_***) of the magnet–inorganic adhesive hybrid fixation module as a function of time is shown in Figure 5f (number of magnets: 3, adhesive area: 100 cm^2^). The total lateral supportive force reached 156 N after 60 s, which is sufficient for the secure attachment of the SBS of 1.4 kg weight on the PEB surface. The supportive force can be modulated by adjusting the number of magnets and adhesive area upon requirement.

### 5.2. Impact Absorber

**Figure 6:**
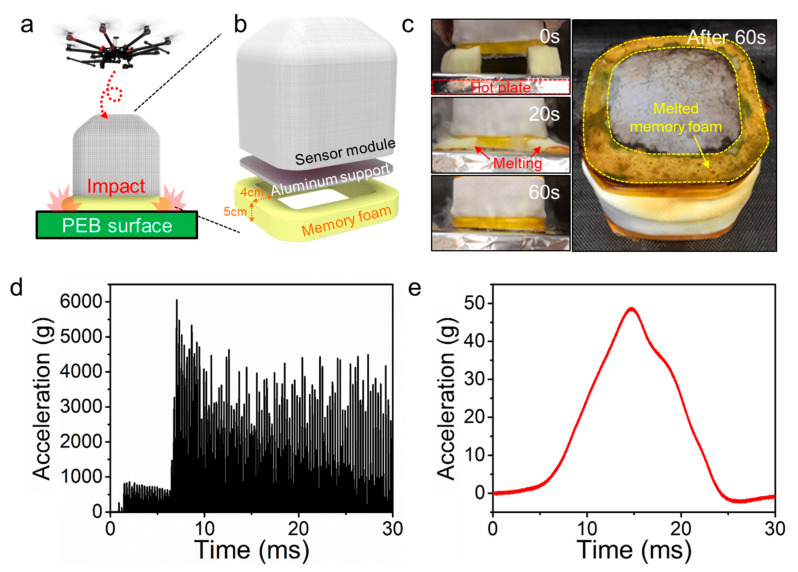
(**a**) Schematic depicting the shock absorbing and sensor deploying mechanism; (**b**) illustration of the installation location and size of the impact absorber; (**c**) glass transition of the memory foam layer on a 500-°C hot plate; (**d**) measured acceleration at the moment of impact without the memory foam cushion layer and (**e**) with the cushion layer.

Most sensors with high sensitivities are vulnerable to not only heat, but also physical impact. In our case, the SBS is transported and dropped over the target location of the PEB roof (Figure 6a). The impact by the collision between the rigid target surface and sensor module can result in fatal failure of the elements of the sensor module and thus needs to be reduced to below the module’s maximum allowable values. However, physical damping should be avoided once the SBS is attached on the PEB because the physical vibration and deformation of the PEB surface must be delivered to the sensor module. To this end, we utilized a viscoelastic polyurethane foam, often referred as a memory form, as an impact absorber due to its desirable properties, such as efficient absorption of impact energy and relatively low glass transition temperature [25,26]. A ring-shaped impact absorber with a width of 4 cm, a height of 5 cm, and a density of 40 kg m^−3^ was attached around the bottom rim of the aluminum support (Figure 6b). When an SBS with the absorber is dropped to the target surface, the absorber reduces the impact energy at the moment of collision. The glass transition and complete melting of the memory foam absorber may occur shortly after the collision due to the high temperature of the PEB surface, which enables an intimate attachment between the aluminum plate of the SBS and the PEB surface; thus, accurate measurements of the vital physical signals from the PEB becomes possible (Figure 6c).

Acceleration at the moment of the impact with and without a memory foam cushion layer was measured using an accelerometer of the sensor module to evaluate the safety of the sensor module elements inside the SBS against physical shock. Among the elements of the sensor modules, the GPS was the most vulnerable part against the physical impact (Table 1); of which, the maximum impact acceleration is 75 g. Thus, the peak impact acceleration at the time of impact should be lower than 75 g. When the SBS was fell at a height of 1 m without a memory foam, the sensor module of the SBS was found to experience a peak impact acceleration of 6056 g (Figure 6d). However, when the shock absorber was installed on the SBS, the peak impact acceleration was significantly reduced to 49 g, indicating that the shock absorber can safely protect the key sensory elements from physical impacts (Figure 6e).

## 6. Testbed Experiments

**Figure 7:**
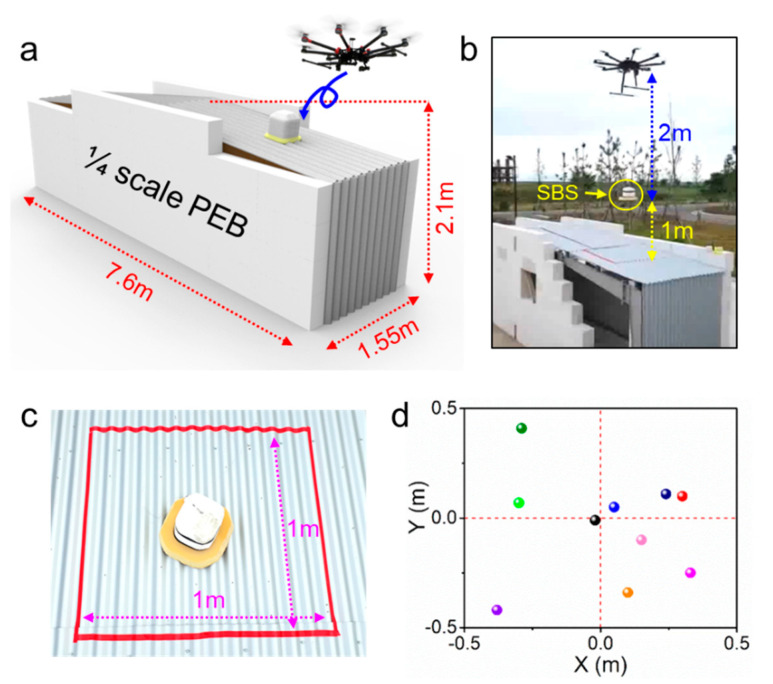
(**a**) Schematic and (**b**) photographic image of the SBS deploying process using a drone during the testbed experiments; (**c**) deployed SBS, dropped from the drone at a height of 1 m; (**d**) positional distribution from 10 trials of the drop-deployment test.

To evaluate overall performance of the developed SBS prototype in a condition reflecting real fire situations, we prepared a 1/4 scale miniaturized PEB structure as a testbed (Figure 7a). First, a remote deployment of the SBS on a target location using a drone was examined (Figure 7b). A SBS prototype was transported by a drone to a height of 3 m above the target point, and the prototype was then lowered to a height of 1 m above the point using a motorized wire. This process was automatically carried out by providing information of the position coordinates to the drone control system. Subsequently, the SBS was released from the drone, and it could be firmly attached to the target point by the aforementioned attachment mechanism. The physical shock at impact could be alleviated by the previously described shock absorber. The positioning error from a target point, which was evaluated from 10 trials of SBS drop tests, was less than 0.3 m (Figure 7c,d. The wind speed during the test was a maximum of 5.4 m s^−1^ and an average of 2.9 m s^−1^.

**Figure 8:**
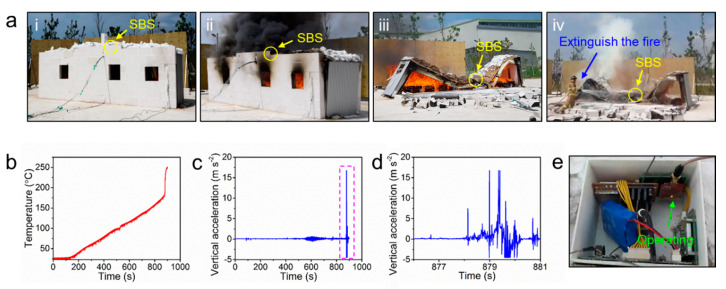
(**a**) Photographs during the testbed experiments; (i) before the fire, (ii) in a fire situation, (iii) after the collapse due to the fire and (iv) extinguishment. Measured values of (**b**) temperature, (**c**) vertical acceleration during the entire testbed experiment and (**d**) at the moment the PEB collapses; (**e**) retrieved sensor module after the test. The sensor module was still operating.

In the next step, we investigated the SHM performance of the SBS by inducing a fire in the testbed PEB (Figure 8a–i). A Fire load for the test was 32.5 kg wood/m^2^ (total 400 kg). The temperature and acceleration data measured by the SBS was wirelessly collected as a function of fire time. The roof temperature after the fire linearly increased and reached 175 °C at 858 s (Figure 8b). This finding indicates that the roof temperature at fire can be lower than the intuitive expectations. However, even at a relatively low roof temperature, structural vibrations were detected by the accelerometer of the SBS (Figure 8c). From 540 s, noticeable, intense peaks were observed in the acceleration data, which indicates that the fire started to induce structural instability in the model PEB. After that, abrupt significant acceleration peaks were detected from 878 s (Figure 8d). This time period was exactly matched with the collapse event of the testbed PEB (Figure 8a–iii), which indicates that the acceleration data of the SBS have potential to be utilized as an important indicator to predict the structural health status. The SBS still worked without any visible damages and malfunction even after the structural collapse and the extinguishment using fire water, as shown in Figure 8a–iv and e, which demonstrates that the SBS is mechanically and physically robust to prevent the key sensing elements from a fire.

## 7. Conclusions

In summary, we have presented a SHM system that can be utilized for monitoring of structural health status in a high-temperature fire environment. The key elements of the sensor system were thermally and mechanically protected by a combinative design using different thermal insulating materials and shock-absorbing materials. The sensor systems were further equipped with a hybrid adhesive module based on magnets and inorganic adhesives. With the integrated device structure, the SBS could be remotely deployed to target locations of the PEB structures without physical damages, firmly adhere to the PEB surface and predict collapse of the structure in a fire. With its light weight (1.4 kg) and compact design (diameter: 20 cm), the SBS could be easily transported to the roof of the PEB by using a drone. The current SBS was designed to work at maximum temperature of 500 °C. However, the maximum allowable working temperature of the SBS could be further increased upon requirements by sacrificing the total weight and size of the system. Although we utilized the SBS for monitoring of the PEB structures, the system can be used as an advanced SHM system for a wider variety of structures and buildings beyond PEB structures based on its advantages, which include facile and immediate device installation, high temperature durability, high sensitivity, wireless data transmission and stable surface attachment. By using this on-demand deployment strategy we expect extensive cost for installation of sensors in pre-existing buildings may be saved, making a SHM system in an emergency more economically affordable and practically accessible.

## Figures and Tables

**Table 1:** Summary of manufacturer, sensing range and limit conditions for each sensor module.

SBS Module	Manufacturer	Sensing Range	Overload Limit (Shock)	Max. Operating Temperature
Accelerometer	PCB piezotronics, 3713E112G	±2 g pk (0.1 mg rms)	±2000 g pk	121 °C
Wi-Fi module	Roving networks, RN-171	1 to 11Mbps	–	85 °C
Power supply	Z-SUN Technology Co., H503450 (3.7 V 900 mA)	–	±150 g pk	60 °C
Thermometer & Thermocouple	Maxim integrated, MAX31855 Sparkfun, Thermocouple K type	−270 to 1372 °C	–	125 °C (Thermometer)
GPS	Swift navigation, Piksi multi	0.01 m +1 ppm	±75 g pk	85 °C
Main module	STMicroelectronics, STM32F767ZIT6 ALTERA, EPM570T100I5 Analog Devices, Inc., AD7779	–	–	85 °C

**Table 2:** Brief properties and characteristics of materials chosen for heat insulation.

Material	Airloy	Pyrogel	Silicate Fabric
Manufacturer	Aerogel Technologies, LLC.	Aspen Aerogels, Inc.	HITCO Carbon Composites, Inc.
Density (g/cm^3^)	0.4	0.2	2.1
Thermal conductivity (W/mK)	0.032	0.025	0.033
Max. operating temperature (°C)	650	300	1593
Compressive modulus (MPa)	113	–	–
Mechanical characteristic	Strong solid	Flexible blanket	Thin fabric
Usage	Insulating support	Insulating filler	Outer cover
